# Care for hospitalized children undergoing institutionalization: distress and defensive strategies of nurses

**DOI:** 10.1590/0034-7167-2024-0529

**Published:** 2026-10-02

**Authors:** Daniela Giotti da Silva, Maria de Lourdes Custódio Duarte, Eduarda Paza Dias, Alessandra Porto d’Ávila, Natália Klauck de Souza, Aline Malaquias de Oliveira, Larissa Gomes de Mattos

**Affiliations:** I Universidade Federal do Rio Grande do Sul. Porto Alegre, Rio Grande do Sul, Brazil

**Keywords:** Child, Hospitalized, Nurses, Pediatric, Child, Foster, Work, Institutionalization., Niño Hospitalizado, Enfermeras Pediátricas, Niño Acogido, Trabajo, Institucionalización.

## Abstract

**Objectives:**

to identify and analyze the factors that generate distress among nurses and the defensive strategies used by them when caring for hospitalized children undergoing institutionalization in a pediatric unit.

**Method:**

a qualitative study conducted in a general hospital in southern Brazil with the participation of 11 nurses. Data were collected through semi-structured interviews and analyzed using thematic analysis from the perspective of Christophe Dejours’ Psychodynamics of Work theoretical framework.

**Results:**

in the first category, social vulnerability, hospitalized children without a companion, and the impact of work settings on personal life were understood by workers as factors of distress. In the second category, the defensive strategies used were conversation groups, patient shift changes, psychotherapy, integrative and complementary therapies, and physical activity.

**Final Considerations:**

this study can help managers promote strategies in the workplace, reducing the psychological burden arising from work and giving new meaning to distress.

## RESUMEN

**Objetivos:**

identificar y analizar los factores que generan sufrimiento en enfermeras y las estrategias defensivas que utilizan al cuidar niños hospitalizados en institucionalización en una unidad pediátrica.

**Métodos:**

estudio cualitativo realizado en un hospital general del sur de Brasil con la participación de 11 enfermeras. Los datos se recopilaron mediante entrevistas semiestructuradas y se analizaron mediante análisis temático desde la perspectiva del marco teórico de la Psicodinámica del Trabajo de Christophe Dejours.

**Resultados:**

en la primera categoría, la vulnerabilidad social, los niños hospitalizados sin acompañante y el impacto del entorno laboral en la vida personal fueron comprendidos por los trabajadores como factores de sufrimiento. En la segunda categoría, las estrategias defensivas utilizadas fueron grupos de conversación, cambios de turno de pacientes, psicoterapia, terapias integrativas y complementarias, y actividad física.

**Consideraciones Finales:**

este estudio puede ayudar a los gerentes a promover estrategias en el lugar de trabajo, reduciendo la carga psicológica derivada del trabajo y resignificando el sufrimiento.

## INTRODUCTION

Hospitalization is considered a stressful and traumatic period for a child, given the separation from their social environment and significant changes to their daily activities. Therefore, they may often face invasive procedures, separation from family and other important people in their lives, making special attention to their emotional well-being necessary^(1)^.

Although hospitalization offers many health benefits to children, this period is accompanied by anxiety and changes, causing psychomotor, affective, and cognitive functions to become fragile due to prolonged hospitalizations, which disrupt daily routines and affect child development^(1)^.

In this context, the presence of a significant person with a child promotes psychological well-being due to the minimization of the negative burden associated with experiencing illness and hospitalization. However, several factors contribute to the absence of an active caregiver for a child during this period, including social vulnerability and a fragile support network due to the lack of a figure who can replace the responsible caregiver, often because the caregiver has other demands. These factors contradict the principles of dignified childcare and alert the healthcare team to the possibility of referring children for preventive and protective measures, even if only temporarily, until family reorganization occurs^(2)^.

In the Brazilian context, the daily experience of social differences can create susceptibility processes that restrict families’ ability to fulfill the basic tasks of supporting and protecting their children. In this regard, considering that families sometimes fail to fulfill their role of providing a safe and conducive environment for child and adolescent development, for various reasons, society and the State need to support these children through assistance, care, and protection at different levels of intervention. Thus, removal from their family unit and institutional care constitute one of the ways to guarantee rights and protect children^(3)^.

It is worth noting that the Child and Adolescent Statute guarantees rights to the infant and juvenile population^(4)^, especially in the institutionalization process, when they are awaiting a decision while still in hospital settings. A hospitalized child undergoing institutionalization is understood to be one who is awaiting a judicial decision regarding institutional care, but who is still hospitalized. Therefore, within hospital settings, demands and needs have been identified for activating the most appropriate measure for each situation, such as social vulnerability, which may prevent a child using devices such as tracheostomy, gastrostomy, ventilator, among others, from returning to their home^(5)^.

Throughout this period, pediatric hospital settings need professionals who are sensitive to children’s psychological and emotional factors so that care is not limited only to their physical needs^(1)^. In this regard, in pediatric units where children receive this referral, nurses can often unintentionally play the role of “substitute”. This happens because the bond created by being the most present professional during hospitalization somehow mitigates the absence of the responsible caregiver^(5)^.

In other words, in addition to possessing theoretical and practical skills, they demonstrate affective involvement in the interpersonal relationships they establish, aiming to contribute to children’s well-being in the pursuit of comprehensive care. Thus, the role played by these workers with these children is compared to that of their own children, with the same concerns and dedication. As a consequence, there is emotional strain, as workers remain attached to children throughout this entire period^(5)^.

Given this perspective, one of the main contributions of Psychodynamics of Work (PoW) is to expose the effects that work organization can have on workers’ mental health, enabling the identification and adoption of preventive measures to understand and intervene in mental health/illness processes in the workplace^(6)^.

One of the concepts proposed by Dejours is distress, which can manifest as dissatisfaction, both physical and psychological, when workers are unable to adapt their job functions to their needs and expectations^(6)^. In this way, workers develop ways to manage these situations in the face of the organizational pressures of daily work, developing defensive strategies related to emotional, affective, and cognitive investments used by workers and their consequences in the sphere of their subjectivity. Thus, when there are no more defensive resources available to workers, a psychological imbalance occurs, causing mental and psychosomatic decompensations^(7)^.

The motivation for this research stems from the authors’ experiences in a pediatric inpatient unit of a general hospital. In this context, it was observed that the interactions of nurses with children undergoing institutionalization often acquired a complexity resembling an “almost parental” relationship, presenting significant and profound overlaps and interconnections.

In relation to the topic, studies were found in the literature on: PoW involving pleasure and distress^(6-9)^; PoW involving only defensive strategies^(10-13)^; children in institutional care and care institutions^(1,3,14)^; and nursing care for hospitalized children^(15,16)^. However, studies addressing the issue of distress and defensive strategies used by nurses in the care of hospitalized children undergoing institutionalization, using PoW framework, are still scarce in the literature, which makes the present study relevant.

Based on these considerations, the following guiding question was sought: what are the factors that generate distress among nurses and the defensive strategies they use when caring for hospitalized children undergoing institutionalization in a pediatric unit? Thus, it is intended that the results of this study will contribute to understanding the specificities of feelings of distress and the defensive strategies used by these workers in light of PoW.

## OBJECTIVES

To identify and analyze the factors that generate distress among nurses and the defensive strategies they use when caring for hospitalized children undergoing institutionalization in a pediatric unit.

## METHODS

### Ethical aspects

The study was an excerpt from a residency completion project entitled “*Prazer e sofrimento do enfermeiro no cuidado à criança hospitalizada em processo de institucionalização em uma unidade pediátrica*”^(5)^. It respected national ethics guidelines, and was approved by the Research Ethics Committee of the proposing institution, under Opinion 5,833,077 (Certificate of Presentation for Ethical Consideration 65904422.8.0000.5327). Participants’ identity was preserved, respecting the items contained in Resolutions 466/12^(17)^ and 510/2016^(18)^ of the Brazilian National Health Council, which provides for ethical standards that regulate research involving human beings.

Personal data was handled in accordance with the General Data Protection Law, which governs the processing of personal data by natural or legal persons, whether public or private, with the aim of protecting the fundamental rights of freedom and privacy, as well as the free development of the personality of the natural person^(19)^. The Informed Consent Form was also provided, read, and explained to each participant. The researcher and the interviewee signed two identical copies.

### Study design

This is a descriptive qualitative study. Qualitative research works with a universe of meanings that corresponds to relationships, processes, and phenomena that cannot be reduced to the operationalization of variables. Furthermore, it aimed to explore the meanings of human actions and relationships, which are not perceptible or capturable in statistics^(20)^.

The research utilized the COnsolidated criteria for REporting Qualitative research guide for manuscript development and writing. Thus, it rigorously met the 32 criteria across the three domains (research team and reflexivity, study concept, and analysis and results), which allowed for its qualification^(21)^.

It is worth noting that the study used PoW as a theoretical framework, and the analyses presented were discussed from the perspective of Christophe Dejours’s writings. He was a French psychiatrist who studied the dynamic relationships between work and the subjective and intersubjective processes that arise from the relationship between the psychological organization of individuals and the work process, justifying the qualitative design based on nurses’ perceptions^(6-8)^.

### Study setting

The study was conducted at a general and university hospital in southern Brazil, in the Pediatric Inpatient Unit belonging to the Pediatric Nursing Service. The unit has 28 beds, consisting of six wards exclusively for the Brazilian Unified Health System, in addition to two semi-private rooms for patients with private health insurance. This unit treats children with chronic diseases and comorbidities, genetic diseases, acute clinical care, and preand post-surgical care.

Since it is considered a semi-critical unit^(22)^, the institution recommends the presence of a health team professional, especially a nurse, full-time in each of the wards. In this way, the unit became a point of reference for children with a history of social vulnerability who, in some cases, are unaccompanied, and the professionals’ presence naturally fosters a closer relationship between the hospitalized children and these workers. This factor influenced the choice of location, as direct and prolonged contact with these patients can generate positive, motivating, and inspiring feelings, or negative ones to the point of making it impossible for professionals to assimilate the potential for causing distress.

### Study participants

The unit has 13 nurses, and all were intentionally invited^(20)^ to participate in the study. Prior to this, the project was presented at a team meeting in the unit’s physical space, where the objectives, purposes and reasons for developing the study were explained. It is worth highlighting that nurses were chosen to be interviewed because these professionals play an important role in care, as they are one of the categories that spends all their time with children at the bedside. Furthermore, they have a leading role in the managerial processes of work organization and care management, justifying the choice of this population.

As mentioned, the unit has 28 beds, where care activities are divided among two nurses per shift (morning, afternoon, and night). In addition to providing care, nurses are also responsible for managerial activities inherent to their practice. Therefore, they participate in team meetings, organize work schedules and staffing levels, as well as develop continuing education activities, which can increase workers’ psychological workload.

Inclusion criteria were established for nurses who had prior experience with children in the institutionalization process, considering a period equal to or greater than six months of work in the pediatric unit, aiming to promote greater familiarity with cases of interest to the study. The exclusion criterion for the study included workers who were on sick leave and maternity leave.

After applying the selection criteria, one nurse was on sick leave for clinical reasons and another nurse had been recently hired, lacking the minimum six months of experience required for inclusion. Therefore, 11 nurses met all the criteria and agreed to participate in the research. At the end of the study, there were no dropouts, and data collection was completed when all eligible workers had been interviewed.

### Data collection and organization

For data collection, invitations were personally extended to the nurses in the unit. Upon confirmation of interest and acceptance, an interview was scheduled outside of working hours, according to each participant’s availability and convenience, taking place before or after care activities.

The data collection instrument was a semi-structured interview containing closed-ended questions about the professionals’ profile (e.g., age, sex, length of service at the institution, length of training) and open-ended questions (Tell me about working with children who are hospitalized in the unit and who will be referred to a foster care institution; Discuss the factors of distress generated when caring for these children; and how do you deal with this? Do you have any strategies for coping with these feelings?). Participants were able to elaborate on the proposed topic and supplement it with more information if they felt it necessary. A pilot test was conducted with a nurse to verify if the questions were appropriate for respondents’ understanding.

Data collection took place in April and May 2023, consisting of 11 interviews lasting between 18 and 26 minutes, conducted by one of the researchers with prior experience in qualitative research data collection. It is important to note that the researchers had no prior interpersonal relationship with participants in order to avoid bias in the study.

The interviews took place in person, in a quiet meeting room at the unit, ensuring privacy. They were recorded and transcribed in full for better analysis and review of the material. At the end, the interviewee was asked if they wished to hear the content of the interview. Thus, only the authors had access to respondents’ answers, which were coded to preserve privacy and maintain anonymity. The professionals interviewed were coded with the letter “W” (Worker), followed by the order in which the interviews took place (e.g., W1, W2...).

### Data analysis

Data were analyzed according to the thematic analysis proposed by Minayo^(20)^, which unfolded in three stages: pre-analysis; material exploration; and treatment of results obtained and interpretation.

The first stage consisted of revisiting the initial research objectives. During this phase, a cursory reading was conducted to allow for direct and intensive contact with the field material, in order to understand the content and construct a *corpus* that corresponds to the studied content in its entirety, resuming the process of the exploratory stage and using as a parameter the exhaustive reading of the material and the initial questions^(20)^.

Also at this stage, recording units, context units (the delimitation of the context of understanding of the recording unit), coding modality and more general theoretical concepts that guided the analysis were determined^(20)^, such as “impotence”, “caregiver without a bond with the child”, “child without a companion”, “vulnerability”, “psychotherapy”, “Reiki”, “meditation”, “physical activity”, “conversation groups in the institution”, and “changing patient schedule”.

The second stage involved grouping the content into categories, aiming to achieve text comprehension. Categorization constitutes a process of text reduction, resulting in data classification and aggregation, separating them into categories responsible for specifying the themes^(20)^, in which the following categories emerged: “Factors generating distress at work when caring for hospitalized children in the institutionalization process”; and “Defensive strategies used by workers when caring for hospitalized children in the institutionalization process”.

Finally, in the third stage, the information obtained was highlighted and, based on it, inferences and interpretations were made^(20)^, relating them around the theoretical dimensions proposed by Dejours and PoW, serving as a theoretical framework for the study^(6-8)^.

## RESULTS

Concerning interviewee characteristics, the age range varied between 26 and 46 years, and all participants were female. As for the length of time workers had been practicing, two nurses had less than five years of experience; eight had between 10 and 20 years; and one had 21 years, being the nurse with the longest period of experience in the study unit during the data collection period. All had postgraduate degrees at the specialization and/or master’s level, and worked 36 hours per week. Furthermore, their length of service in the pediatric inpatient unit ranged from six months to 25 years.

### Factors generating distress at work when caring for hospitalized children in the institutionalization process

Based on interviews with nurses from the study unit, the following subcategories emerged regarding factors that generate distress when caring for a hospitalized child undergoing institutionalization: “Social vulnerability”; “Hospitalized child without a companion”; and “Difficulty in separating personal and professional relationships”.

The vulnerabilities faced by the families of hospitalized children undergoing institutionalization are complex, as conditions for supporting a child dependent on technology are often precarious at home.

*Some have social and economic problems involved* [...]. *We end up getting involved with the issues of the parents, the family, the child, because it’s a child who is in a precarious social environment and still has many needs, dependent on health technologies that they can take home* [...]. *When we go to make the home visit, there’s no electricity, no basic sanitation, and then they have to go to the shelter.* (W4)

*Sometimes, the family takes good care of the child, but then they have to go home with a feeding tube or tracheostomy, and therefore they are unable to provide care at home and the child will be institutionalized.* (W5)

Healthcare workers frequently encounter barriers related to the vulnerabilities of the child and their family, interfering with preand post-hospital care. Thus, during interviews, nurses stated that one of the situations that generates the most distress is when children are placed in institutional care, as children’s home environment is incompatible with their care needs.

In the unit studied, not all children undergoing institutionalization are unaccompanied, as the reasons for institutionalization are multiple and complex, such as precarious living conditions in highly vulnerable contexts. However, workers refer to children who do not have companions during hospitalization as a source of distress due to a lack of empathy for patients’ reality.

*How unhelpful that situation is, because she’s not our daughter, she’s her mother’s daughter. But since she found herself alone, everyone became a bit like a mother, father, brother, in other words, her family.* (W8)

*When a child cries asking for a family member and we call, but the person doesn’t come, it causes distress to know that a child is distressed, because we do everything we can, but obviously we are not the family.* (W10)

Workers reported distress when caring for these unaccompanied children, since they end up taking on the role of mother because they feel responsible for providing affection. Even though all the professionals involved in the care welcome children, these are limited moments restricted to workday procedures and do not replace family members, especially when a child misses these people.

Another factor that generates distress, according to interviewees, is that work settings impacts their personal lives. In other words, it became evident that nurses had difficulty dealing with the excessive attachment demanded by the situation experienced in that environment, generating an emotional burden for workers.

*We end up blurring the lines a bit with the professional nursing role. I, at least, have difficulty maintaining that boundary* [...]*. We create an extra bond. But sometimes I think it ends up getting in the way; not everyone can separate and process this situation in a more objective way. For those* [children] *who stay longer, it’s more complicated for us and for them.* (W2)

*There are children with whom we form a bond that transcends the professional relationship, because there is affection and a desire for the best to happen. And sometimes, what we think would be best for that child doesn’t happen, and this generates a lot of frustration in the team. Everything was assessed, several professionals were involved, and they considered that this decision* [institutional care] *was the best one.* (W5)

Interviewees reported negative feelings, such as frustration at certain times, due to difficulties with excessive bonding time with children, since the hospitalization period is prolonged and they feel responsible for children, increasing the psychological burden of work. Furthermore, nurses’ private lives are invaded by feelings of responsibility and anxieties outside of working hours, demonstrating the challenge of establishing boundaries in this relationship.

### Defensive strategies used by workers when caring for hospitalized children in the institutionalization process

Based on interviews with nurses from the study unit, the following subcategories emerged regarding the defensive strategies used by workers when caring for a hospitalized child undergoing institutionalization: conversation groups; patient shift changes; psychotherapy; integrative and complementary therapies; and physical activity.

The focus groups within the institution were strategies that the interviewees mentioned as a way to alleviate the stress arising from caring for these children. They reiterated the importance of dialogue among the team as a way to obtain support and unity in the face of distressing situations that occur at work.

*When we have a group session here with the nurses, I try to bring up certain topics for us to discuss, because I think that when you talk, you can get a lot out. Not everyone uses other resources like physical exercise or therapy to find themselves.* (W2)

*I think expressing yourself, talking to colleagues helps a lot* [...] *talking about feelings is very important, because often we don’t talk about them. We talk about the situation, but we distance ourselves from it. It’s important to talk about feelings and allow yourself to feel them.* (W5)

*We end up doing group therapy amongst ourselves. I think it’s important to talk to your colleagues so we know how to act, what to say, how to approach things. So even the approach itself is a painful process for us; we find it quite difficult. But being able to work on this issue with your team is also very important.* (W9)

According to the statements, nurses recognize the importance of communication among team members, creating collective spaces for speaking and listening in a protected environment and finding social and emotional support through their relationships with colleagues. In this way, this strategy makes work settings more bearable by allowing for collective coping, giving new meaning to the work experienced and reframing distress.

Caring for children in the institutionalization process demands a high level of psychological strain from workers. Therefore, changing work schedules was also identified by nurses as a coping strategy for the distress they experience in this work context.

*There was a nursing technician who was assigned to bed 21 and frequently presented medical certificates. We started talking and he was suffering a lot because of the patients he cared for, and I said, “Maybe you could take a break from this schedule for a while”. We can organize things. Few people realize it. There are many people who are emotionally unwell. No matter how much we try to lighten the mood, we know it’s a stressful and hostile environment.* (W2)

*I think changing shifts helps because there won’t be contact, it will try to avoid the situation, and if I’m not feeling well at that moment, it will make me feel worse. One way to protect myself is by changing shifts.* (W3)

Nurses state that changing the work schedule of professionals who are emotionally overwhelmed is a strategy to avoid absenteeism and work-related illnesses. Therefore, it is essential for nurses to have this perspective regarding their team, and they can use this method to help workers maintain well-being at work. Thus, nurses’ professional relationships extend beyond direct care of children and include interaction with other nursing professionals. They must also recognize the risks that the demands of that child’s context may pose to their mental health and that of their team.

In addition to collective strategies, workers use psychotherapy to alleviate the distress generated by caring for these children. This tool has had positive effects on interviewees, demonstrating the importance of therapeutic support in dealing with and coping with work-related issues that spill over into personal life.

*There was a time when I needed therapy to avoid getting so involved. My desire was to go with the patient and try to solve the problem. I would be with my family on a Sunday, and I would talk about the patient and cry. It was a lot of distress; I think therapy was very good. Nowadays, I can deal with it calmly.* (W1)

*Sometimes I’ve worked in therapy on feelings, the transition itself for a patient who spent eight years here; it was a very difficult thing to let go of* [...] *work is part of our lives, it’s hard to separate. I end up setting those limits, but I also have cases that I discuss in therapy. Especially those children who are left alone for long periods, where we end up replacing the family at various times.* (W2)

*I’ve had to see a psychologist before because it was getting too heavy. But I think it’s really important for us to try to preserve our mental health. Before, I kept a lot to myself, I had crying spells, I was sad, I was withdrawn. But later, with professional help, I learned that I have to talk about feelings, I have to experience them, because the more you suppress them, somehow, they will come out. Sometimes it’s in the form of illness, sometimes it’s muscle pain. I had a lot of muscle pain.* (W5)

Therapy helps interviewees recognize their limits so that the psychological burden resulting from their work does not become exhausting, preventing repercussions on both mental and physical health, such as muscle pain or illness. Furthermore, the psychotherapy setting provides a safe space to verbalize the feelings generated by caring for these children, helping them cope with a reality full of challenges.

Another strategy recognized by female workers for its effects on promoting and maintaining physical and mental health is the practice of integrative and complementary therapies and physical activity as a way to alleviate the tensions generated in work settings.

*I meditate, I want to get back into yoga, I use flower essences, I use other non-pharmacological techniques to relieve tension. I exercise every week and for me it works as a way to de-stress. I use these options as a way to release stress.* (W2)

*I take flower essences, I’ve done acupuncture, Reiki, family constellation therapy-they’re complementary things-so having a belief, believing in something, asking for strength on those days when you’re not feeling so well, so you can arrive here* [at work] *feeling good and give your best. It’s good to be rested, have a good diet, and a good night’s sleep.* (W7)

Workers’ statements demonstrated that both integrative and complementary therapies and the practice of physical activity are strategies that help in coping with challenging situations in their daily work, as positive effects are perceived in maintaining their mental health and promoting their well-being. Furthermore, it is observed that engaging in other activities reduces the psychological burden arising from work, making it lighter and more tolerable.

Work is not limited to the physical space where the act of working takes place, but extends to the subjectivity of each worker. Therefore, these professionals seek to modify aspects of their daily work, resorting to individual and collective defensive strategies in order to confront and alleviate the distress generated, according to the PoW framework.

## DISCUSSION

In the context of pediatric hospitalization, nurses work with children hospitalized for various reasons; however, children who need to be referred to foster care institutions due to social vulnerability increase the psychological burden on workers, as they are unable to change the children’s reality. Furthermore, feelings of hopelessness can worsen professionals’ state of desolation, as children will be referred to foster care services^(16)^ until the family is reorganized. Therefore, these situations demand emotional preparedness from nurses at the time of children’s discharge, since they need to detach themselves from all the physical and psychological burden built up until then, generating a feeling of belonging and insecurity for no longer being responsible for the care^(14)^.

This vulnerability of hospitalized children often stems from the social, economic, and emotional conditions of families that can prolong hospitalization^(23)^, resulting in greater emotional involvement on the part of workers in these cases. Some caregivers may need to leave their jobs due to the need to accompany the hospitalized child, causing financial difficulties for the family or even leaving the child unaccompanied, due to the impossibility of leaving their job.

In this setting, hospitalized children without a companion can generate greater distress for workers, who end up having, in their work, attitudes that go beyond their professional sphere, increasing the psychological burden and generating stress. In these prolonged hospitalizations, the hospital replaces the coexistence of home and family with health professionals, since they only interact with the workers, due to interrupted family ties^(14)^, which can generate psychological distress in these nurses.

In environments such as the one studied, one can observe a state of distress resulting from an overload of work or a very high psychological burden^(6)^. In addition to this, workers rarely leave their state of alert and stress, whether due to seeing the children unaccompanied or because the children express longing for a caregiver who is not expected to visit them.

In this way, hospitalization in childhood disrupts a child’s routine, causing a stressful time in their lives, and when there is no family member present during this process, the situation becomes even more challenging^(1)^. Thus, in an attempt to create a more welcoming environment for children, workers end up crossing the fine line between the professional and personal boundaries.

When distress occurs in the process of identifying with a child, there is a tendency to associate one’s own perspective on how one would act in a given situation. Therefore, the feelings and attitudes that guide professional practice and performance are particular to each individual, making it impossible to separate the personal and professional worlds^(9)^.

Similarly, the difficulty in separating personal aspects from professional life also has repercussions in the family sphere, since work is inherent to people’s daily lives, and bringing home worries and distress surrounding the child who was cared for becomes inevitable. Thus, it is like wanting to separate work from the other pieces that make up the “puzzle” of each worker’s life^(9)^.

Healthcare professionals are often encouraged to regulate their own emotions and tolerate the pressure and frustrations of daily life. Therefore, when these workers are unable to abstract aspects related to work settings using their creativity, the “psychological discharge pathway” becomes blocked, accumulating this energy and becoming a source of tension and displeasure^(6)^.

In this setting, defensive strategies emerge. These refer to a concept formulated by Dejours, which signifies a psychological mediation by workers to confront, or not be aware of, work-related distress, changing their perception in relation to organizational pressures. In this confrontation, defense strategies can be individual or collective. Individual strategies are mechanisms that workers use, often unconsciously, to rationalize or deny their own distress, regardless of the presence of other people. Collective strategies, on the other hand, refer to how workers organize themselves when there is a common goal, i.e., to reduce the psychological burden that work imposes on them^(6)^.

These defense mechanisms can be considered transformative, since they transcend what is experienced by healthcare workers, allow for the construction of a new shared perspective, and develop new cognitive processes in the face of situations that generate distress at work^(7)^. The use of these strategies is beneficial for workers and “unintentional”, as it occurs when an individual appropriates their own distress to reframe it, overcome it, and transform it into pleasure. In this way, it becomes an anticipatory action on the concrete conditions of work organization, considered the individuals’ emancipation^(12)^.

Working means dealing with moments of tension while sharing a common space, confronting the imperfection of reality, and building collective goals^(7)^. This understanding indicates that it is common for professionals to seek social support, turning to colleagues when difficulties and problems are work-related, as these are generally common to everyone in this context^(10)^. Thus, dialogue within the team tends, in practice, to alleviate distress and reduce the psychological burden on workers.

In spaces for discussion and reflection, it is possible to establish sharing, which leads to a differentiated way of coping with and transforming the organization of work. Thus, strengthening these mechanisms can help maximize collective strategies and, consequently, transform distress into cooperation within the team^(11)^. For PoW, workers’ struggle to cope with the psychological distress resulting from an inflexible organization requires them to use their creativity and subjective intelligence, reducing physical and mental exhaustion^(24)^.

Therefore, it is necessary to pay attention to aspects of the service’s operation, such as adjusting work schedules and monitoring professionals so that they are supported and encouraged, aiming to increase their level of satisfaction. By easing this workload, there is a positive impact by reducing absenteeism due to fatigue and high stress levels^(24)^.

Nurses understand that there is a close relationship between work and the process of illness among professionals and that, often, they are physically present at work, but are unable to fully perform their work activities due to psychological decompensation. As a result of the impact of this care, there is a need to pay attention to the reorganization of work schedules, as children in the process of institutionalization may increase the psychological workload^(25)^, due to the emotional involvement and bond developed throughout the hospitalization period.

In this context, collective defense strategies often help individuals cope with a reality they could not endure alone, using only individual strategies^(8)^. Furthermore, they promote the organization, cohesion, and stabilization of work groups, and their operation can be interrupted when the individuals involved no longer desire it^(7)^.

Furthermore, professionals seek individual strategies, which can take the form of social and cultural practices, often combined with relationships with others, as they help workers to rationalize distress through a dynamic that makes sense to them outside of work^(12)^. Therefore, one strategy used by workers for self-knowledge is psychotherapy, which can encourage the learning process for coping with everyday situations, reducing the psychological burden arising from work. The search for specialized professional support, such as psychologists and psychiatrists, and the use of medication for mental health have increased in recent years, being one of the supports that reduce work-related distress^(13)^.

Other individual defensive strategies workers employ to avoid stress and improve working conditions include non-pharmacological practices, such as integrative and complementary health therapies, and physical activity. The fact that interviewees seek activities outside work settings may be positive in minimizing distress, although it expresses a compensation closer to defense, since these strategies avoid contact with the distress arising from work situations, without seeking changes in the causes of distress, but rather ways to control it^(12)^.

Physical activity, essential for human beings, reduces professional stress, and its regular practice is among the psychosocial factors that decrease the risk of mental illness, in addition to protecting and promoting workers’ overall health. Furthermore, rest and adequate nutrition are other strategies used to help prevent mental and physical health problems^(26)^.

The use of integrative and complementary therapies has also become, over the years, a practice for health prevention and promotion through comprehensive care, with the potential to increase people’s coping capacity in the face of distress. Strategies such as yoga, Reiki, meditation, aromatherapy, therapeutic music, and acupuncture can be defense mechanisms for reducing the psychological burden on workers^(27)^. These mechanisms should not be seen as alienation from labor, but as a temporary defense mechanism that will strengthen workers in an attempt to transform distress into pleasure^(12)^.

The different strategies presented by workers, as well as the predominance of the use of individual coping mechanisms, indicate the psychological effort they make to mediate the job demands. The need to use individual defense strategies is exercised by workers to balance the demands of work processes, aiming to reduce discomforts arising from labor^(11)^.

Therefore, it is necessary to establish programs within the healthcare institution that welcome workers and provide social support, aiming at the development of healthy coping strategies. Actions that value connection and/or affection can be the foundation for promoting worker care in the workplace, providing spaces for sharing perceptions and experiences, where people have moments to speak and listen^(11)^.

### Study limitations

It is important to emphasize that the present research had limitations, such as the selection of only one pediatric inpatient unit from a single hospital. This is because there is a possibility that workers from other units that also receive children with the studied profile may have different perceptions of factors that generate distress and defensive strategies to alleviate them.

### Contributions to nursing, health, or public policy

The study contributed to the discussion of issues related to worker health in the studied location, as it brought visibility to the distress of this category and triggered a process of reflection on the topic in light of PoW. Furthermore, by understanding the strategies used by these workers, managers can apply this knowledge to create spaces that promote health in the workplace, reducing the resulting psychological burden and attempting to give new meaning to distress.

## FINAL CONSIDERATIONS

This study made it possible to analyze the factors that cause distress to nurses who care for hospitalized children undergoing institutionalization in a pediatric unit, and the defensive strategies they use to alleviate the psychological burden arising from their work.

Factors causing distress arise when nurses face the complexities of caring for hospitalized children without a companion, which can occur for various reasons, including aspects of vulnerability in relation to families. This setting weakens the bond and attachment between professionals and children, leading to the intrusion of work-related situations into the personal lives of these nurses.

As for coping strategies, workers used different mechanisms to alleviate the psychological burden generated by caring for these children. In work settings, they use dialogue with colleagues in groups and manage work schedules in order to reduce the distress of the routine. Furthermore, in addition to collective strategies, workers sought other individual forms of support, such as psychotherapy, physical activity, and complementary therapies, in pursuit of a better quality of life.

This study revealed that work has repercussions both in workers’ professional lives and personal lives, and the use of strategies is often a way to control and reduce the psychological burden, since the profile of hospitalized children in these care settings can continue to reproduce feelings of distress and stress in these professionals. Considering the care of children in the institutionalization process, it is important to encourage new studies that encourage the perspective of other professionals on the health team, in order to broaden the understanding of distress generated by work and the possibilities of strategies used by these workers, aiming to alleviate the psychological burden and stress of work settings, in line with the PoW framework.

## Data Availability

The research data are available within the article.
