# Supplementary material for: Care for hospitalized children undergoing institutionalization: distress and defensive strategies of nurses
Source: Rev Bras Enferm. 2026 Oct 2;79:e20240529. doi: 10.1590/0034-7167-2024-0529 (PMC13631968; doi:10.1590/0034-7167-2024-0529)
Supplement: Translation

# Translation: Cuidado às crianças hospitalizadas em processo de institucionalização: sofrimento e estratégias defensivas de enfermeiros

**DOI:** 10.1590/0034-7167-2024-0529pt

**Authors:** Daniela Giotti da Silva, Maria de Lourdes Custódio Duarte, Eduarda Paza Dias, Alessandra Porto d’Ávila, Natália Klauck de Souza, Aline Malaquias de Oliveira, Larissa Gomes de Mattos

**Affiliations:** I Universidade Federal do Rio Grande do Sul. Porto Alegre, Rio Grande do Sul, Brasil

**Keywords:** Criança Hospitalizada, Enfermeiros Pediátricos, Criança Acolhida, Trabalho, Institucionalização.

## Abstract

**Objetivos:**

identificar e analisar os fatores geradores de sofrimento dos enfermeiros e as estratégias defensivas utilizadas por eles ao cuidar de crianças hospitalizadas em processo de institucionalização em uma unidade pediátrica.

**Métodos:**

estudo qualitativo, realizado em hospital geral do sul do Brasil, com participação de 11 enfermeiras. As informações foram coletadas através de entrevista semiestruturada e analisadas conforme análise temática sob a ótica do referencial teórico da Psicodinâmica do Trabalho de Christophe Dejours.

**Resultados:**

na primeira categoria, vulnerabilidade social, criança hospitalizada sem acompanhante e repercussão do ambiente laboral na vida pessoal foram entendidos pelas trabalhadoras como fatores de sofrimento. Já na segunda categoria, as estratégias defensivas utilizadas foram grupos de conversa, troca de escalas de pacientes, psicoterapia, terapias integrativas e complementares, e atividade física.

**Considerações Finais:**

este estudo pode auxiliar gestores a promoverem estratégias no ambiente laboral, diminuindo a carga psíquica advinda do trabalho e ressignificando o sofrimento.

## INTRODUÇÃO

A hospitalização é considerada um período estressante e traumático para a criança, considerando o distanciamento do seu meio social e modificações significativas das suas atividades cotidianas. Dessa maneira, não raramente ela pode se deparar com procedimentos invasivos, afastamento da convivência familiar e de outras pessoas importantes em sua vida, sendo necessário especial atenção para seu aspecto emocional^(1)^.

Apesar de a hospitalização propiciar muitos benefícios à saúde da criança, esse período vem acompanhado de ansiedade e mudanças, fazendo com que as funções psicomotoras, afetivas e cognitivas fiquem fragilizadas devido a internações prolongadas, que interrompem o cotidiano e afetam o desenvolvimento infantil^(1)^.

Nesse contexto, a presença de uma pessoa significativa afetivamente junto à criança promove o bem-estar psicológico, devido à minimização da carga negativa associada à vivência de doenças e sua hospitalização. Entretanto, são diversos os fatores que acarretam a ausência de participação ativa de um acompanhante para a criança nesse período, incluindo vulnerabilidade social e rede de apoio frágil por falta de uma figura que possa substituir o cuidador responsável, muitas vezes por este ter outras demandas. Esses fatores vão de encontro com os pressupostos de um cuidado digno à criança e alertam a equipe de saúde para a possibilidade de encaminhamento à aplicação de medidas preventivas e de proteção, mesmo que provisoriamente, até a reorganização familiar^(2)^.

No cenário brasileiro, a vivência cotidiana de situações de diferenças sociais pode configurar processos de susceptibilidade que restringem as possibilidades das famílias de cumprirem as tarefas básicas de amparo e proteção aos seus filhos. Nesse sentido, considerando que, por vezes, famílias não conseguem exercer seu papel de proporcionar um ambiente seguro e propício ao desenvolvimento infantojuvenil, por motivos distintos, a sociedade e o Estado precisam amparar essa criança através de apoio, cuidado e proteção em diferentes níveis de intervenção. Assim, o afastamento do seu núcleo familiar e acolhimento institucional se configuram uma das formas de garantia de direitos e proteção à criança^(3)^.

Cabe ressaltar que o Estatuto da Criança e do Adolescente assegura direitos à população infantojuvenil^(4)^, sobretudo no processo de institucionalização, quando ela aguarda a decisão ainda no cenário hospitalar. Entende-se por criança hospitalizada em processo de institucionalização aquelas que estão aguardando a decisão judicial no que se refere ao acolhimento institucional, mas que ainda estão hospitalizadas. Sendo assim, no cenário hospitalar, foram identificadas demandas e necessidades de acionamento da medida mais assertiva para cada situação, como vulnerabilidade social, o que pode impedir a criança em uso de dispositivos como traqueostomia, gastrostomia, respirador, entre outros, retornar ao seu domicílio^(5)^.

Durante todo esse período, o ambiente hospitalar pediátrico precisa contar com profissionais sensíveis aos fatores psíquicos e emocionais das crianças, de modo que o cuidado não se restrinja apenas às suas necessidades físicas^(1)^. Nesse sentido, nas unidades pediátricas que as crianças recebem esse encaminhamento, o enfermeiro, muitas vezes, involuntariamente pode assumir a figura de “substituição”. Isso acontece porque a vinculação gerada por ser o profissional mais presente durante a hospitalização, de alguma forma, ameniza a ausência do cuidador responsável^(5)^.

Ou seja, além de serem detentores de competências teórico-práticas, evidenciam o envolvimento afetivo nas relações interpessoais que estabelecem, com vistas a contribuir para o bem-estar da criança na busca pela integralidade do cuidado. Dessa forma, o papel desempenhado pelos trabalhadores com essas crianças é comparado com o de seus próprios filhos, com as mesmas preocupações e dedicação. Como consequência, há desgaste emocional, pois o mesmo se encontra vinculado à criança durante todo esse período^(5)^.

Tendo em vista esta perspectiva, uma das principais contribuições da Psicodinâmica do Trabalho (PDT) é a de expor os efeitos que a organização do trabalho pode gerar na saúde mental do trabalhador, possibilitando a identificação e a adoção de medidas preventivas para compreender e intervir nos processos de saúde/doença mental no ambiente laboral^(6)^.

Um dos conceitos propostos por Dejours é o sofrimento, que pode se expressar em insatisfação, tanto de natureza física quanto de psíquica, no momento em que o trabalhador não é capaz de adaptar as funções laborais às suas necessidades e expectativas^(6)^. Dessa maneira, os trabalhadores elaboram formas de manejar essas situações, perante as pressões organizacionais do cotidiano laboral, com o desenvolvimento de estratégias defensivas relacionadas a investimentos emocionais, afetivos e cognitivos utilizados pelos trabalhadores e suas consequências na esfera de sua subjetividade. Assim, quando não há mais recursos defensivos a serem utilizados pelos trabalhadores, ocorre um desequilíbrio psíquico, causando descompensações mentais e psicossomáticas^(7)^.

A motivação para esta pesquisa é derivada das vivências das autoras em uma unidade de internação pediátrica de um hospital geral. Nesse contexto, percebeu-se que as interações dos enfermeiros para com as crianças em processo de institucionalização muitas vezes adquiriam uma complexidade que se assemelhava a uma relação “quase parental”, apresentando sobreposições e entrelaçamentos significativos e profundos.

Em relação ao tema, foram encontrados na literatura estudos sobre: PDT envolvendo prazer e sofrimento^(6-9)^; PDT envolvendo apenas as estratégias defensivas^(10-13)^; crianças em processo de acolhimento institucional e instituições de acolhimento^(1,3,14)^; e cuidado de enfermagem com crianças hospitalizadas^(15,16)^. No entanto, estudos que abordem a temática do sofrimento e das estratégias defensivas utilizadas por enfermeiros no cuidado de crianças hospitalizadas em processo de institucionalização, utilizando o referencial da PDT, ainda são escassos na literatura, o que torna o presente estudo relevante.

A partir dessas considerações, buscou-se responder à seguinte questão norteadora: quais os fatores geradores de sofrimento dos enfermeiros e as estratégias defensivas utilizadas por eles ao cuidar de crianças hospitalizadas em processo de institucionalização em uma unidade pediátrica? Assim, pretende-se que os resultados deste estudo auxiliem no conhecimento das especificidades diante dos sentimentos de sofrimento e as estratégias defensivas utilizadas por esses trabalhadores à luz da PDT.

## OBJETIVOS

Identificar e analisar os fatores geradores de sofrimento dos enfermeiros e as estratégias defensivas utilizadas por eles ao cuidar de crianças hospitalizadas em processo de institucionalização em uma unidade pediátrica.

## MÉTODOS

### Aspectos éticos

O estudo foi um recorte de trabalho de conclusão de residência intitulado “Prazer e sofrimento do enfermeiro no cuidado à criança hospitalizada em processo de institucionalização em uma unidade pediátrica”^(5)^. Respeitou as diretrizes de ética nacionais, sendo aprovado pelo Comitê de Ética em Pesquisa da instituição proponente, sob Parecer nº 5.833.077 (Certificado de Apresentação para Apreciação Ética nº 65904422.8.0000.5327). Aos participantes, foi assegurada a preservação da identidade, sendo respeitados os itens que constam nas Resoluções nº 466/12^(17)^ e nº 510/2016^(18)^ do Conselho Nacional de Saúde, que dispõe sobre as normas éticas que regulamentam a pesquisa envolvendo seres humanos.

Os dados pessoais foram manejados em conformidade com a Lei Geral de Proteção de Dados Pessoais, que dispõe sobre o tratamento de dados pessoais por pessoa natural ou jurídica, de direito público ou privado, com o objetivo de proteger os direitos fundamentais de liberdade e de privacidade, bem como o livre desenvolvimento da personalidade da pessoa natural^(19)^. Também foi fornecido, lido e elucidado para cada participante o Termo de Consentimento Livre e Esclarecido. A pesquisadora e o entrevistado assinaram duas cópias de mesmo teor.

### Tipo de estudo

Trata-se de estudo qualitativo do tipo descritivo. A pesquisa qualitativa trabalha com um universo de significados que corresponde às relações, processos e fenômenos que não podem ser reduzidos à operacionalização de variáveis. Além disso, visa explorar os significados das ações e relações humanas, o que não é perceptível e captável em estatísticas^(20)^.

A pesquisa utilizou o guia *COnsolidated criteria for REporting Qualitative research* para desenvolvimento e escrita do manuscrito. Assim, atendeu rigorosamente aos 32 critérios dos três domínios (equipe de pesquisa e reflexividade, conceito do estudo, e análise e resultados), o que oportunizou a sua qualificação^(21)^.

Cabe ressaltar que o estudo utilizou a PDT como referencial teórico, e as análises tecidas foram discutidas sob a ótica dos escritos de Christophe Dejours. Ele foi um médico psiquiatra francês que estudou as relações dinâmicas entre o trabalho e os processos subjetivos e intersubjetivos que surgem da relação entre a organização psíquica das pessoas com o processo laboral, justificando o delineamento qualitativo a partir das percepções dos enfermeiros^(6-8)^.

### Cenário do estudo

O estudo foi realizado em hospital geral e universitário do Sul do Brasil, na Unidade de Internação Pediátrica pertencente ao Serviço de Enfermagem Pediátrica. A unidade possui 28 leitos, sendo constituída por seis enfermarias destinadas exclusivamente ao Sistema Único de Saúde, além de dois quartos semiprivativos destinados aos convênios. Nesta unidade, ocorrem hospitalizações de crianças com doenças e comorbidades crônicas, doenças genéticas, cuidados clínicos agudos, prée pós-cirúrgicos.

Por ser considerada uma unidade semicrítica^(22)^, a instituição preconiza a permanência de um profissional da equipe de saúde, em especial o enfermeiro, em tempo integral, em cada uma das enfermarias. Dessa maneira, a unidade se tornou uma referência para crianças com histórico social de vulnerabilidade que, em alguns casos, estão desacompanhadas, e essa permanência do profissional naturalmente gera uma aproximação entre as crianças hospitalizadas e esses trabalhadores. Tal motivo influenciou a escolha do local, pois o contato direto e prolongado com esses pacientes pode gerar sentimentos positivos, motivadores e inspiradores, ou negativos, a ponto de impossibilitar o profissional de assimilar o potencial gerador de sofrimento.

### Participantes do estudo

A unidade possui 13 enfermeiros, e todos foram convidados de forma intencional^(20)^ para participar do estudo. Previamente, ocorreu a apresentação do projeto em reunião de equipe no espaço físico da unidade, onde foram expostos os objetivos, propósitos e razões para o desenvolvimento do estudo. Cabe ressaltar que se optou por entrevistar os enfermeiros, devido ao fato de que esses profissionais assumem papel importante na assistência, uma vez que compõem uma das categorias que convivem com a criança à beira-leito em tempo integral. Além disso, possuem protagonismo nos processos gerenciais de organização do trabalho e gestão do cuidado, justificando a escolha dessa população.

Como mencionado, a unidade dispõe de 28 leitos, onde as atividades assistenciais são divididas em dois enfermeiros por turno (manhã, tarde e noite). Além da assistência, o enfermeiro também se encarrega de atividades gerenciais que são inerentes à sua *práxis*. Dessa forma, participa de reuniões de equipe, organiza escalas de trabalho e o dimensionamento de pessoal, bem como o desenvolvimento de atividades de educação permanente, o que pode gerar aumento da carga psíquica do trabalhador.

Foram estabelecidos critérios de inclusão para enfermeiros que tivessem experiência prévia com crianças em processo de institucionalização, considerando um período igual ou maior de seis meses de atuação na unidade pediátrica, visando promover maior familiarização com casos de interesse do estudo. O critério de exclusão para o estudo abrangeu os trabalhadores que estivessem em licença saúde e licença-maternidade.

Após a aplicação dos critérios de seleção, um enfermeiro se encontrava em licença saúde por questões clínicas e um enfermeiro havia sido recém-contratado, não havendo uma experiência mínima de seis meses para ser incluído. Sendo assim, 11 enfermeiras atenderam a todos os critérios e aceitaram participar da pesquisa. Ao final do estudo, não houve desistências, e a coleta de dados foi encerrada quando todos os trabalhadores elegíveis foram entrevistados.

### Coleta e organização dos dados

Para a coleta de dados, foram feitos convites pessoalmente aos enfermeiros da unidade. Após ter ido confirmado o interesse e devido aceite, foi agendada entrevista em um horário fora da jornada de trabalho, conforme disponibilidade e comodidade de cada participante, ocorrendo préou pós-atividades assistenciais.

O instrumento de coleta de dados foi entrevista semiestruturada contendo questões fechadas sobre o perfil dos profissionais (como idade, sexo, tempo de trabalho na instituição, tempo de formação) e questões abertas (Fale sobre o trabalho com crianças que estão hospitalizadas na unidade e que serão encaminhadas para instituição de acolhimento; Fale sobre fatores de sofrimento gerados ao cuidar dessas crianças; e Como você lida com isso? Possui alguma estratégia para lidar com esses sentimentos?). Os participantes puderam discorrer sobre o tema proposto e complementar com mais informações, caso achassem necessário. Realizou-se um teste piloto com um enfermeiro para verificar se as questões estavam adequadas para a compreensão dos entrevistados.

A coleta das informações foi realizada nos meses de abril e maio de 2023, contando com 11 entrevistas de duração entre 18 e 26 minutos, que foram aplicadas por uma das pesquisadoras com experiência prévia em coleta de pesquisa qualitativa. Vale ressaltar que as pesquisadoras não tinham relação interpessoal prévia com os participantes, a fim de não gerar viés no estudo.

As entrevistas ocorreram de maneira presencial, em uma sala de reuniões da unidade, sem ruídos e com garantia da privacidade, sendo gravadas e transcritas na íntegra para melhor análise e leitura do material. Ao final, foi questionado sobre a vontade do entrevistado de ouvir o conteúdo da entrevista. Nesse sentido, somente as autoras tiveram acesso às respostas dos entrevistados, as quais foram codificadas para preservar a privacidade e manter o anonimato. As profissionais entrevistadas foram codificadas pela letra “T” (Trabalhadora), seguido pela ordem em que as entrevistas ocorreram (ex., T1, T2...).

### Análise dos dados

Os dados foram analisados de acordo com a análise temática proposta por Minayo^(20)^, que se desdobrou em três etapas: pré-análise; exploração do material; e tratamento dos resultados obtidos e interpretação.

A primeira etapa consistiu na retomada dos objetivos iniciais da pesquisa. Durante essa fase, foi realizada leitura flutuante para contato direto e intenso com o material de campo, a fim de compreender o conteúdo e constituir um *corpus*, que corresponde ao conteúdo estudado em sua totalidade, retomando o processo da etapa exploratória e tendo como parâmetro a leitura exaustiva do material e as indagações iniciais^(20)^.

Ainda nessa fase, determinaram-se as unidades de registro, as unidades de contexto (a delimitação do contexto de compreensão da unidade de registro), a modalidade de codificação e os conceitos teóricos mais gerais que orientaram a análise^(20)^, tais como “impotência”, “cuidador sem vínculo com a criança”, “criança sem acompanhante”, “vulnerabilidade”, “psicoterapia”, “Reiki”, “meditação”, “atividade física”, “grupos de conversa na instituição” e “trocar a escala de pacientes”.

A segunda etapa incluiu o agrupamento de categorias onde o conteúdo foi organizado, visando alcançar a compreensão do texto. A categorização se constitui em um processo de redução do texto, resultando na classificação e agregação de dados, separando-os em categorias responsáveis pela especificação dos temas^(20)^, em que emergiram as seguintes categorias: “Fatores geradores de sofrimento no trabalho ao cuidar de crianças hospitalizadas em processo de institucionalização”; e “Estratégias defensivas utilizadas pelos trabalhadores ao cuidar de crianças hospitalizadas em processo de institucionalização”.

Por fim, na terceira etapa, evidenciaram-se as informações obtidas e, a partir dela, foram realizadas inferências e interpretações^(20)^, relacionando-as em torno das dimensões teóricas propostas por Dejours e PDT, servindo como referencial teórico para o estudo^(6-8)^.

## RESULTADOS

Em relação à caracterização das entrevistadas, a faixa etária variou entre 26 e 46 anos, e todas as participantes foram do sexo feminino. Quanto ao tempo de formação das trabalhadoras, duas enfermeiras possuíam menos de cinco anos de formação; oito possuíam entre 10 e 20 anos; e uma possuía 21 anos, sendo a enfermeira com maior tempo de formação na unidade do estudo no período da coleta de dados. Todas possuíam pós-graduação em nível de especialização e/ou mestrado, e trabalhavam em regime de 36 horas semanais. Além disso, o tempo de trabalho na unidade de internação pediátrica variou entre seis meses e 25 anos.

### Fatores geradores de sofrimento no trabalho ao cuidar de crianças hospitalizadas em processo de institucionalização

A partir das entrevistas com as enfermeiras da unidade do estudo, emergiram as seguintes subcategorias sobre fatores geradores de sofrimento ao cuidar de uma criança hospitalizada em processo de institucionalização: “vulnerabilidade social”; “criança hospitalizada sem acompanhante”; e “dificuldade de separar a relação pessoal e profissional”.

As vulnerabilidades apresentadas pelas famílias das crianças hospitalizadas em processo de institucionalização são complexas, pois, muitas vezes, as condições de manter uma criança dependente de tecnologias são precárias no domicílio.

*Algumas têm problemas sociais e econômicos envolvidos* [...]. *A gente acaba se envolvendo com as questões dos pais, da família, da criança, porque é uma criança que está no meio social precário e ainda cheio de demandas, dependente de tecnologias em saúde para ele levar para a casa* [...]. *Quando vamos fazer a visita domiciliar não tem luz, não tem saneamento básico, e aí precisa ir para o abrigo.* (T4)

*Às vezes, a família cuida bem da criança, mas vai ter que voltar para casa com sonda ou com traqueostomia, daí não tem condições de cuidado em casa e vai ser institucionalizada por isso.* (T5)

Não raramente, os trabalhadores de saúde encontram barreiras que estão relacionadas às vulnerabilidades da criança e de sua família, interferindo no cuidado prée pós-hospitalar. Assim, as enfermeiras verbalizaram, durante as entrevistas, que uma das situações que mais geram sofrimento é quando ocorre o acolhimento institucional infantil, pois a criança apresenta um contexto domiciliar incompatível com as suas demandas de cuidado.

Na unidade estudada, não são todas as crianças em processo de institucionalização que estão desacompanhadas, pois os motivos de acolhimento institucional são múltiplos e complexos, como a própria precarização domiciliar em contextos de grande vulnerabilidade. Entretanto, as trabalhadoras referem-se às crianças que não possuem acompanhantes durante a hospitalização como um fator de sofrimento pela empatia devido à realidade dos pacientes.

*O quanto aquilo ali não é benéfico porque ela não é filha da gente, ela é filha da mãe dela. Mas como ela se viu sozinha, todo mundo se tornou um pouco mãe, pai, irmão, ou seja, a família dela.* (T8)

*Quando a criança chora pedindo familiar e a gente liga, mas a pessoa não vem, então gera sofrimento saber que a criança está sofrendo, porque a gente faz toda a parte que a gente pode, mas obviamente não somos a família.* (T10)

As trabalhadoras referiram sofrimento ao cuidar dessas crianças desacompanhadas, uma vez que elas acabam assumindo a maternagem por se sentirem responsáveis em proporcionar afeto. Por mais que todos os profissionais envolvidos no cuidado acolham a criança, são momentos limitados e restritos aos procedimentos da jornada de trabalho e que não substituem os membros da família, sobretudo quando a criança sente saudade dessas pessoas.

Outro fator que gera sofrimento, de acordo com as entrevistadas, é que a relação do ambiente laboral repercute na vida pessoal. Ou seja, ficou evidente a dificuldade de as enfermeiras lidarem com a vinculação excessiva demandada pela situação vivenciada nesse ambiente, gerando uma carga emocional para as trabalhadoras.

*A gente acaba misturando um pouco a relação do profissional enfermeiro. Eu pelo menos tenho dificuldade de manter este limite* [...]*. A gente cria um vínculo extra. Mas algumas vezes eu acho que acaba atrapalhando, nem todo mundo consegue separar, processar de uma maneira mais objetiva esta situação. Essas* [crianças] *que ficam mais tempo é mais complicado para gente e para elas.* (T2)

*Tem crianças que cria um vínculo e perpassa a relação profissional, porque tem carinho e quer sempre que o melhor aconteça. E às vezes, não acontece o que a gente acha que seria o melhor para aquela criança e isso gera muita frustração na equipe. Tudo foi avaliado, vários profissionais foram envolvidos e consideraram que essa decisão* [acolhimento institucional] *foi a melhor.* (T5)

As entrevistadas referem sentimentos negativos, como a frustração em determinados momentos, devido a dificuldades com excessivo tempo de vinculação com a criança, uma vez que o tempo de hospitalização é prolongado e elas se sentem responsáveis pela mesma, aumentando a carga psíquica do trabalho. Além disso, o espaço da vida privada das enfermeiras é invadido pelo sentimento de responsabilidade e anseios fora da jornada de trabalho, demonstrando o desafio de estabelecer limites nessa relação.

### Estratégias defensivas utilizadas pelos trabalhadores ao cuidar de crianças hospitalizadas em processo de institucionalização

A partir das entrevistas com as enfermeiras da unidade do estudo, emergiram as seguintes subcategorias sobre as estratégias defensivas utilizadas pelas trabalhadoras ao cuidar de uma criança hospitalizada em processo de institucionalização: grupos de conversa; troca de escalas de pacientes; psicoterapia; terapias integrativas e complementares; e atividade física.

Os grupos de conversa na instituição foram estratégias que as entrevistadas referiram como uma maneira de amenizar o estresse advindo do cuidado prestado a essas crianças. Elas reiteram a importância do diálogo entre a equipe como forma de se obter suporte e união diante das situações geradoras de sofrimento que ocorrem no trabalho.

*Quando a gente faz um grupo aqui das enfermeiras, eu tento trazer alguns assuntos para tentarmos conversar, porque acho que quando tu falas, consegue colocar muita coisa para fora. Nem todo mundo usa outros recursos como exercício físico, terapia para se reconhecer.* (T2)

*Acho que expressar, falar com colegas ajuda muito* [...] *falar sobre os sentimentos é muito importante, porque muitas vezes, a gente não fala. Falamos da situação, mas nos afastamos dela. É importante falar sobre os sentimentos e se permitir sentir.* (T5)

*A gente acaba fazendo terapia de grupo entre nós. Eu acho importante conversar com teus colegas para sabermos como agir, o que falar, como abordar. Então até a abordagem é um processo doloroso para nós, a gente encontra bastante dificuldade nisso. Mas tu poder trabalhar essa questão com a tua equipe, é muito importante também.* (T9)

De acordo com os depoimentos, as enfermeiras reconhecem a importância da comunicação entre os membros da equipe, criando espaços coletivos de fala e escuta em um ambiente protegido e encontrando suporte social e emocional por meio da relação com os colegas. Dessa maneira, essa estratégia torna a realidade laboral mais suportável ao permitir o enfrentamento coletivo, dando novo sentido ao trabalho vivenciado e ressignificando o sofrimento.

O cuidado às crianças em processo de institucionalização exige alta carga psíquica das trabalhadoras. Sendo assim, a troca de escala de trabalho também foi apontada pelas enfermeiras como uma estratégia de enfrentamento ao sofrimento diante desse contexto laboral.

*Tinha um técnico de enfermagem que ficava fixo no leito 21 e seguidamente apresentava atestados. Começamos a conversar e ele sofria muito por causa dos pacientes que atendia e eu disse: “Quem sabe fica um pouco fora dessa escala”. A gente tem como organizar. São poucas as pessoas que se dão conta. Tem muita gente doente emocionalmente. Por mais que a gente descontraia, sabemos que é um lugar de estresse e um ambiente hostil.* (T2)

*Eu acho que a troca de escala ajuda, porque não vai ter o contato, vai tentar evitar a situação, se eu não estou bem naquele momento vai me fazer ficar pior. Uma maneira de me proteger é trocando a escala.* (T3)

As enfermeiras referem que trocar a escala de trabalho do profissional que está sobrecarregado emocionalmente é uma estratégia para evitar o absenteísmo e o adoecimento relacionado ao ambiente laboral. Desse modo, é essencial esse olhar do enfermeiro em relação à sua equipe, podendo utilizar esse método para auxiliar os trabalhadores na manutenção do bem-estar no trabalho. Nesse sentido, as relações profissionais do enfermeiro extrapolam o cuidado direto à criança e incluem a interação com outros profissionais da enfermagem. Ele também deve reconhecer os riscos que as demandas do contexto daquela criança podem impactar sua saúde mental e a de sua equipe.

Além das estratégias coletivas, as trabalhadoras utilizam a psicoterapia para amenizar o sofrimento gerado ao cuidar dessas crianças. Essa ferramenta gerou efeitos positivos nas entrevistadas, demonstrando a importância do acompanhamento terapêutico para lidar e enfrentar questões da rotina de trabalho que atravessam a vida pessoal.

*Teve uma época que eu precisei fazer terapia, para não ficar tão envolvida. A minha vontade era ir junto com o paciente e tentar resolver. Eu estava em família, no domingo, e eu falava do paciente e chorava. Era um sofrimento muito grande, acho que a terapia foi muito boa. Hoje em dia eu consigo lidar tranquilo com isso.* (T1)

*Algumas vezes eu trabalhei na terapia sobre sentimentos, a própria transição de uma paciente que ficou oito anos aqui dentro, foi uma coisa bem difícil de desapegar* [...] *o trabalho faz parte da vida da gente, é difícil separar. Eu acabo dando esses limites, mas também tenho casos que discuto na terapia. Principalmente essas crianças que ficam muito tempo desacompanhadas, que a gente acaba substituindo a família em vários momentos.* (T2)

*Já precisei procurar a psicóloga, porque estava muito pesado. Mas acho que é bem importante a gente tentar preservar a nossa saúde mental. Antes eu guardava muito para mim, tinha crise de choro, ficava triste, ficava retraída. Mas depois, com ajuda profissional, eu aprendi que tenho que falar sobre sentimentos, tenho que vivenciar ele, porque quanto mais sufoca, de alguma forma ele vai sair. Às vezes é em doença, às vezes é dor muscular. Eu tinha muita dor muscular.* (T5)

A terapia auxilia as entrevistadas a reconhecerem seus limites, para que a carga psíquica decorrente da atividade laboral não seja exaustiva, evitando repercussões tanto na saúde mental quanto na saúde física, por meio de dores musculares ou doenças. Ademais, o espaço da psicoterapia fornece um local seguro para verbalizar os sentimentos gerados no cuidado a essas crianças, auxiliando no enfrentamento de uma realidade repleta de desafios.

Outra estratégia reconhecida pelas trabalhadoras por seus efeitos na promoção e manutenção da saúde física e mental é a prática de terapias integrativas e complementares e de atividade física como forma de amenizar as tensões geradas no ambiente de trabalho.

*Eu faço meditação, ioga quero voltar, eu uso florais, eu utilizo outras técnicas não farmacológicas para aliviar as tensões. Faço exercício físico toda semana e para mim funciona como uma forma de desestressar. Eu uso essas opções como válvula de escape.* (T2)

*Eu tomo floral, já fiz acupuntura, Reiki, constelação familiar, são coisas complementares, então ter uma crença, acreditar em alguma coisa, pedir força naqueles dias que tu não tá tão bem, pra poder chegar bem aqui* [trabalho] *e dar o meu melhor. É bom que a gente esteja descansada, tenha uma boa alimentação, um bom sono.* (T7)

As falas das trabalhadoras demonstraram que tanto as terapias integrativas e complementares quanto a prática de atividade física são estratégias que auxiliam no enfrentamento de situações desafiadoras do seu cotidiano de trabalho, pois são percebidos os efeitos positivos na manutenção de sua saúde mental e promoção do seu bem-estar. Além disso, observa-se que se ocupar com outras atividades diminui a carga psíquica advinda do labor, tornando-o mais leve e tolerável.

O trabalho não se esgota no espaço físico onde se executa o ato de trabalhar, mas na subjetividade de cada trabalhador. Sendo assim, as profissionais buscam modificar aspectos do seu cotidiano laboral, recorrendo a estratégias defensivas individuais e coletivas, a fim de enfrentar e amenizar o sofrimento gerado, segundo o referencial da PDT.

## DISCUSSÃO

No contexto da internação pediátrica, os enfermeiros trabalham com hospitalizações infantis por diferentes motivos, porém crianças que necessitam ser encaminhadas para instituições de acolhimento devido à vulnerabilidade social geram o aumento de carga psíquica do trabalhador, por ele não conseguir mudar a realidade dessa criança. Além disso, o sentimento de desesperança pode agravar o quadro de desolação do profissional, pois a criança será encaminhada para serviços de acolhimento^(16)^ até que haja reorganização da família. Dessa forma, essas situações demandam um preparo emocional do enfermeiro no momento da alta da criança, uma vez que ele precisa se desvincular de toda a carga física e psíquica construída até então, gerando um sentimento de despertencimento e insegurança por não ser mais o responsável pelo cuidado^(14)^.

Essa vulnerabilidade das crianças hospitalizadas advém, muitas vezes, de uma condição social, econômica e emocional das famílias que pode prolongar a internação^(23)^, ocasionando maior envolvimento afetivo no trabalhador nesses casos. Alguns acompanhantes podem precisar se afastar dos seus vínculos empregatícios pela necessidade de acompanhamento da criança hospitalizada, acarretando dificuldades financeiras para a família ou até mesmo deixando a criança desacompanhada, devido à impossibilidade de afastar-se de seu emprego.

Nesse cenário, as crianças hospitalizadas sem acompanhante podem gerar maior sofrimento nos trabalhadores, que acabam assumindo, em seu trabalho, atitudes que vão além da sua esfera profissional, aumentando a carga psíquica e gerando estresse. Nessas internações prolongadas, o hospital substitui a convivência de casa e dos familiares pelos profissionais de saúde, uma vez que elas passam a interagir apenas com os trabalhadores, em razão de vínculo familiar interrompido^(14)^, podendo gerar sofrimento psíquico nesses enfermeiros.

Em ambientes como o local estudado, pode-se observar um estado de sofrimento resultante de uma sobrecarga de trabalho ou de uma carga psíquica muito elevada^(6)^. Somado a isso, os trabalhadores dificilmente abandonam o estado de alerta e estresse, seja pelo fato de ver a criança desacompanhada ou por elas verbalizarem saudade de um cuidador que não tem previsão de visitá-las.

Dessa maneira, a hospitalização na infância rompe com a rotina da criança, ocasionando um momento estressante na vida delas, e quando não há acompanhamento de um familiar durante esse processo, o cenário se torna ainda mais desafiador^(1)^. Assim, na tentativa de o trabalhador promover um ambiente mais acolhedor para a criança, ele acaba atravessando a linha tênue entre o limite profissional e o pessoal.

Quando o sofrimento ocorre no processo de identificação do profissional com a criança, há uma tendência de associar a própria ótica de como agiria em determinada situação. Dessa forma, os sentimentos e as atitudes que norteiam o exercício e o desempenho profissional são particulares de cada indivíduo, não sendo possível separar o mundo pessoal do laboral^(9)^.

Do mesmo modo, a dificuldade em separar aspectos pessoais da vida profissional também emite reflexos no âmbito familiar, pois o trabalho é inerente ao cotidiano das pessoas, e levar para a casa preocupações e sofrimentos em torno da criança que foi atendida torna-se inevitável. Assim, é como querer separar o trabalho das demais peças que compõem o “quebra-cabeça” da vida de cada trabalhador^(9)^.

Os profissionais de saúde são frequentemente incentivados a regular as suas próprias emoções, tolerar a pressão e as frustrações da vida cotidiana. Assim, quando esse trabalhador não consegue abstrair aspectos relacionados ao ambiente laboral utilizando-se sua criatividade, a “via de descarga psíquica” fica fechada, acumulando essa energia e tornando-se fonte de tensão e desprazer^(6)^.

Nesse cenário, surgem as estratégias defensivas. Elas se referem a um conceito formulado por Dejours, que significa uma mediação psíquica do trabalhador para enfrentar ou não ter consciência do sofrimento laboral, mudando sua percepção em relação às pressões organizacionais. Nesse enfrentamento, as estratégias de defesa podem ser individuais ou coletivas. As estratégias individuais são mecanismos que os trabalhadores utilizam, muitas vezes de modo inconsciente, para racionalizar ou negar o próprio sofrimento, independentemente da presença de outras pessoas. Já as coletivas se referem à maneira como os trabalhadores se organizam quando há um objetivo em comum, ou seja, diminuir a carga psíquica que o trabalho lhes impõe^(6)^.

Esses mecanismos de defesa podem ser considerados transformadores, uma vez que transcendem aquilo que é vivido pelos trabalhadores da saúde, permitem a construção de um novo ponto de vista compartilhado e elaboram novos processos cognitivos diante das situações geradoras de sofrimento no trabalho^(7)^. O uso dessas estratégias é benéfico para os trabalhadores e “não intencional”, pois ocorre quando o sujeito se apropria do próprio sofrimento para ressignificá-lo, superá-lo e transformá-lo em prazer. Dessa forma, torna-se ação antecipada sobre as condições concretas da organização do trabalho, sendo considerada a emancipação do sujeito^(12)^.

Trabalhar é lidar com momentos de tensão ao compartilhar um espaço em comum, enfrentar a imperfeição do real e constituir objetivos no coletivo^(7)^. Tal entendimento indica que é habitual que os profissionais procurem por apoio social, recorrendo aos colegas quando as dificuldades e problemas estão relacionados ao trabalho, pois geralmente são comuns a todos nesse cenário^(10)^. Assim, o diálogo entre a equipe tende a fornecer, na prática, a função de atenuar o sofrimento e diminuir a carga psíquica do trabalhador.

Em espaços de discussão e reflexão, é possível estabelecer a partilha, o que leva à forma diferenciada de enfrentamento e de transformação da organização do trabalho. Assim, o fortalecimento desses dispositivos poderá auxiliar na maximização das estratégias coletivas e, consequentemente, na transformação do sofrimento em cooperação entre a equipe^(11)^. Para a PDT, a luta do trabalhador para lidar com o sofrimento psíquico, decorrente de uma organização inflexível, requer que ele faça uso de sua criatividade e inteligência subjetiva, diminuindo a exaustão física e mental^(24)^.

Dessa maneira, é necessário que haja atenção para aspectos do funcionamento do serviço, como adequação de escalas de trabalho e acompanhamento dos profissionais, para que sejam apoiados e incentivados, objetivando aumentar seu nível de satisfação. Ao amenizar essa carga de trabalho, há um impacto positivo por diminuir a ocorrência de absenteísmo em decorrência do cansaço e do alto nível de estresse^(24)^.

Os enfermeiros entendem que há uma estreita relação entre o trabalho e o processo de adoecimento dos profissionais e que, muitas vezes, os mesmos estão fisicamente presentes no trabalho, porém não conseguem desempenhar plenamente suas atividades laborais, devido às descompensações psíquicas. Em decorrência do impacto desse cuidado, há a necessidade de atentar para a reorganização das divisões das escalas de trabalho, pois crianças em processo de institucionalização podem aumentar a carga psíquica laboral^(25)^, devido ao envolvimento emocional e vínculo desenvolvido ao longo do período de hospitalização.

Nessa conjuntura, frequentemente, as estratégias coletivas de defesa contribuem para que o indivíduo suporte uma realidade que não conseguiria suportar isoladamente, fazendo somente o uso de estratégias individuais^(8)^. Ademais, favorecem a organização, a coesão e a estabilização dos coletivos de trabalho, e podem ter a sua atuação interrompida quando os indivíduos envolvidos não a desejarem mais^(7)^.

Além disso, os profissionais buscam estratégias individuais, que podem se constituir em práticas sociais e culturais, frequentemente somadas às relações com os outros, pois auxiliam o trabalhador a racionalizar o sofrimento por meio de uma dinâmica que faça sentido para ele, fora do labor^(12)^. Dessa forma, uma estratégia utilizada pelos trabalhadores para o autoconhecimento é a psicoterapia, que é capaz de estimular o processo de aprendizagem para o enfrentamento de situações cotidianas, diminuindo a carga psíquica advinda do trabalho. A busca por apoio profissional especializado, como psicólogos e psiquiatras, e o uso de medicamentos para a saúde mental têm aumentado nos últimos anos, sendo um dos suportes que reduzem o sofrimento laboral^(13)^.

Outras estratégias defensivas individuais dos trabalhadores para evitar o estresse e melhorar as condições no trabalho incluem práticas não farmacológicas, como terapias integrativas e complementares em saúde, e atividade física. O fato de as entrevistadas buscarem atividades fora do ambiente laboral pode ser positivo para minimizar o sofrimento, embora expresse uma compensação mais próxima da defesa, pois essas estratégias evitam contato com o sofrimento advindo das situações de trabalho, sem buscar mudanças nas causas do sofrimento, mas formas de controlá-lo^(12)^.

A atividade física, essencial para o ser humano, diminui o estresse do profissional, e sua prática regular se encontra entre os fatores psicossociais que diminuem o risco para o adoecimento psíquico, além da proteção e promoção da saúde integral dos trabalhadores. Ademais, o descanso e a alimentação adequada são outras estratégias utilizadas que ajudam na prevenção de problemas de saúde mental e física^(26)^.

O uso de terapias integrativas e complementares também se tornou, ao longo dos anos, uma prática para a prevenção e promoção da saúde por meio de um cuidado integral, com potencial de aumentar a capacidade de enfrentamento das pessoas diante do sofrimento vivenciado. Estratégias como yoga, Reiki, meditação, aromaterapia, música terapêutica e acupuntura podem ser mecanismos de defesa para a diminuição da carga psíquica do trabalhador^(27)^. Esses mecanismos não devem ser tomados como alienação do trabalho, mas como defesa momentânea que vai fortalecer o trabalhador na tentativa de transformar o sofrimento em prazer^(12)^.

As diferentes estratégias apresentadas pelas trabalhadoras, assim como o predomínio da utilização de enfrentamento individual, indicam o esforço psíquico que elas fazem para mediar as demandas do trabalho. A necessidade de uso das estratégias de defesa individuais é exercida pelos trabalhadores para equilibrar as exigências dos processos de trabalho, visando diminuir os desconfortos advindos do labor^(11)^.

Dessa maneira, é necessário estabelecer programas dentro da instituição de saúde que acolham o trabalhador e forneçam apoio social, visando o desenvolvimento de estratégias de enfrentamento saudáveis. Ações que valorizem o vínculo e/ou o afeto podem ser o alicerce para promoção de cuidado dos trabalhadores no ambiente laboral, propiciando espaços para compartilhamento de percepções e experiências, em que as pessoas tenham momentos para falar e ouvir^(11)^.

### Limitações do estudo

É importante ressaltar que a presente pesquisa teve limitações, como a escolha de apenas uma unidade de internação pediátrica de um único hospital. Isso porque há a possibilidade de os trabalhadores de outras unidades que também recebem crianças com o perfil estudado terem diferentes percepções de fatores geradores de sofrimento e estratégias defensivas para amenizá-los.

### Contribuições para a área da enfermagem, saúde ou políticas públicas

O estudo contribuiu para a discussão de questões relacionadas à saúde do trabalhador do local estudado, pois deu visibilidade ao sofrimento dessa categoria e desencadeou um processo de reflexão sobre o tema à luz da PDT. Além disso, ao conhecer as estratégias utilizadas por esses trabalhadores, os gestores podem aplicar esse conhecimento na criação de espaços que promovam a saúde no ambiente de trabalho, diminuindo a carga psíquica decorrente dele e tentando ressignificar o sofrimento.

## CONSIDERAÇÕES FINAIS

Com este estudo, pôde-se analisar os fatores que geram sofrimentos às enfermeiras que cuidam de crianças hospitalizadas em processo de institucionalização em uma unidade pediátrica e as estratégias defensivas utilizadas por elas para amenizar a carga psíquica advinda do trabalho realizado.

Os fatores de sofrimento surgem a partir do momento em que as trabalhadoras se deparam com a complexidade do cuidado à criança hospitalizada sem acompanhante, que pode ocorrer por diversos motivos, incluindo aspectos de vulnerabilidade em relação às famílias. Esse cenário causa um estreitamento das relações de vínculo e apego da profissional com a criança, gerando a intromissão das situações do ambiente de trabalho na vida pessoal dessas enfermeiras.

Já em relação às estratégias defensivas, as trabalhadoras utilizaram diferentes mecanismos para amenizar a carga psíquica gerada a partir do cuidado a essas crianças. No contexto do ambiente laboral, elas utilizam o diálogo com colegas em grupo e gerenciam as escalas de trabalho, a fim de diminuir o sofrimento da rotina. Ademais, além das estratégias coletivas, as trabalhadoras buscaram outras formas individuais, como psicoterapia, atividade física e terapias complementares, em busca de qualidade de vida.

Com este estudo, percebeu-se que o trabalho gera repercussão tanto na própria vida laboral quanto no espaço pessoal dos trabalhadores, e a utilização de estratégias, muitas vezes, é uma forma de controlar e diminuir a carga psíquica, uma vez que o perfil de crianças hospitalizadas nesses espaços de cuidado pode continuar reproduzindo sentimentos de sofrimento e estresses nesses profissionais. Considerando o cuidado à criança em processo de institucionalização, é importante o incentivo a novos estudos que instiguem a ótica dos demais profissionais da equipe de saúde, a fim de ampliar o entendimento sobre sofrimento gerado pelo trabalho e as possibilidades de estratégias utilizadas por esses trabalhadores, visando amenizar a carga psíquica e o estresse do ambiente laboral, indo ao encontro do referencial da PDT.

## Data Availability

Os dados de pesquisa estão disponíveis no corpo do artigo.
